# Sacituzumab Govitecan initial dose reduction in polish patients with metastatic triple-negative breast cancer: impact on efficacy and safety

**DOI:** 10.1007/s00280-025-04823-3

**Published:** 2025-10-04

**Authors:** Małgorzata Pieniążek, Marcin Kubeczko, Manuela Las-Jankowska, Anna Polakiewicz-Gilowska, Aleksandra Łacko, Michał Jarząb, Zuzana Bielčiková, Renata Pacholczak-Madej, Miroslawa Püsküllüoğlu

**Affiliations:** 1 Department of Oncology, Wrocław Medical University, Plac Hirszfelda 12, Wrocław, 53-413 Poland; 2 Lower Silesian Comprehensive Cancer Center, Plac Hirszfelda 12, Wrocław, 53-413 Poland; 3 Breast Cancer Unit, Gliwice Branch, Maria Skłodowska-Curie National Research Institute of Oncology, Gliwice, 44-102 Poland; 4 Surgical Oncology, Oncology Centre, Ludwik Rydygier Collegium Medicum in Bydgoszcz, Nicolaus Copernicus University in Torun, Bydgoszcz, 85-067 Poland; 5 Department of Clinical Oncology, Oncology Center-Prof Franciszek Lukaszczyk Memorial Hospital, Bydgoszcz, 85-796 Poland; 6 Department of Oncology, First Faculty of Medicine, Charles University, General University Hospital, Prague, Czech Republic; 7 Department of Gynecological Oncology, Maria Sklodowska-Curie National Research Institute of Oncology, Garncarska Street 11, 31-115 Krakow, Branch, Poland; 8 Department of Anatomy, Jagiellonian University Medical College, Świętej Anny 12, Krakow, 31-008 Poland; 9 Department of Clinical Oncology, Kraków Branch, Maria Sklodowska-Curie National Research Institute of Oncology, Kraków, Poland

**Keywords:** Sacituzumab govitecan, Triple negative breast cancer, Metastases, Dose reduction, Real world data

## Abstract

**Background:**

Sacituzumab govitecan (SG) is approved for metastatic triple-negative breast cancer in ≥ 2 line setting at 10 mg/kg IV on Days 1 and 8 (21-day cycle). Trials confirmed its superiority over 8 mg/kg with manageable safety. In practice, precautionary dose reductions are used despite no formal guidance. In Poland, fixed 200 mg vials and unreimbursed drug waste lead to early dose adjustments.

**Methods:**

This retrospective study evaluated the impact of initial SG dose reduction on treatment outcomes and tolerability in Polish patients. Medical records provided data on baseline features, treatment, survival, and safety. Kaplan-Meier and chi-square tests were used for survival and group comparisons. A multivariate Cox model assessed the independent effect of dose reduction on overall survival (OS) and progression-free survival (PFS). Significance was set at *p* < 0.05.

**Results:**

Among 83 patients (median age 55, range 30–86), initial dose reductions ≥ 10% were observed in 16 patients (19.3%), including 9 (10.8%) with dose reduced ≥ 20%. Administrative adjustments (reductions > 10% to flat doses of 200 mg multiplications) accounted for 18.1% of the entire cohort. Grade ≥ 2 and ≥ 3 adverse events occurred in 83.1% and 56.6%, respectively. In a multivariate analysis, a ≥ 20% initial dose reduction remained an independent predictor of shorter PFS (HR: 2.6; 95% CI: 1.1–6.6; *p* = 0.04) and OS (HR: 6; 95% CI: 2–17.5; *p* = 0.001). Initial dose reduction did not affect toxicity.

**Conclusions:**

In this preliminary report initial dose reduction of SG negatively impacted PFS and OS without reducing toxicity, highlighting the need for further studies and dosing policy adjustments.

**Supplementary Information:**

The online version contains supplementary material available at 10.1007/s00280-025-04823-3.

## Introduction

Triple-negative breast cancer (TNBC) represents a biologically aggressive breast cancer subtype characterized by the absence of estrogen receptor (ER), progesterone receptor (PR), and human epidermal growth factor receptor 2 (HER2) expression [1, 2]. Due to the lack of actionable targets, systemic treatment options remain limited, particularly in the advanced setting. In this context, sacituzumab govitecan (SG) has emerged as a valuable therapeutic option for patients with metastatic TNBC who have progressed after standard therapies [1–3]. Information on the safety, efficacy, dosing strategies, and premedication practices with SG derived from clinical daily practice is particularly important in light of recent data demonstrating that primary endpoints have been met in clinical trials evaluating SG in the first-line palliative setting. The phase III ASCENT-04/KEYNOTE-D19 trial evaluated SG in combination with pembrolizumab versus chemotherapy plus pembrolizumab in previously untreated, programmed death-ligand 1 (PD-L1)–positive (combined positive score [CPS] ≥ 10) locally advanced or metastatic TNBC. SG plus pembrolizumab significantly improved progression-free survival with a favorable safety profile, supporting its potential as a new frontline standard [4]. These findings suggest a forthcoming regulatory approval in this setting, likely expanding the population of patients eligible for SG-based treatment [4].

Sacituzumab govitecan (SG) is a first-in-class antibody-drug conjugate (ADC) targeting trophoblast cell surface antigen 2 (TROP-2), a transmembrane glycoprotein frequently overexpressed in epithelial malignancies. The construct includes a humanized IgG1κ monoclonal antibody specifically targeting TROP-2, linked to the cytotoxic agent SN-38 via a cleavable linker. SN-38, a topoisomerase I inhibitor derived from irinotecan, is conjugated at a high drug-to-antibody ratio of approximately 7.6. After intravenous administration, SG binds to TROP-2 and is internalized, releasing SN-38 in lysosomes to induce DNA damage and apoptosis. Through a bystander effect, SN-38 also affects neighboring cells, enhancing efficacy in heterogeneous TNBC [5–9].

In the first-in-human trial, SG was evaluated in pretreated patients with advanced solid tumors [10]. SG was administered on days 1 and 8 of 21-day cycles, with dose escalation from 8 to 18 mg/kg. Neutropenia was the primary dose-limiting toxicity [10]. Although 12 mg/kg was identified as the maximum tolerated dose for the first cycle, it proved too toxic with repeated administration. Lower doses (8 and 10 mg/kg) were better tolerated, enabling prolonged treatment. Given the observed objective responses and favorable therapeutic index at 10 mg/kg, this dose was selected for subsequent clinical development [11].

In line with these early findings, subsequent phase II and III trials—as well as the approved summary of product characteristics (SmPC)—established 10 mg/kg of body weight as the standard dosing regimen for SG [12]. The drug is administered intravenously on days 1 and 8 of each 21-day cycle. The initial infusion is given over 3 h, with the duration reduced to 1–2 h in subsequent cycles if no infusion-related reactions occur. Patients should be monitored closely during the infusion and for at least 30 min afterward to detect potential infusion-related adverse events [12].

The aim of this study was to assess how initial dose reductions of SG influence treatment outcomes and safety in patients with metastatic TNBC treated in routine clinical settings in Poland. In particular, the analysis focused on the effects of administering ≤ 80% of the recommended starting dose on overall survival, progression-free survival, and the incidence of adverse events. The study also considered the practical and systemic factors contributing to early dose modifications, such as reimbursement limitations and fixed vial sizes, which may lead to precautionary reductions not supported by clinical guidelines.

## Materials and methods

### Patient selection and data collection

The study enrolled patients with inoperable or metastatic TNBC. ER and PR negativity was defined as nuclear staining in less than 1% of invasive tumor cells. HER2 expression was assessed by immunohistochemistry (IHC; score 0–3): score 0 indicated no or faint, incomplete staining in ≤ 10% of cells; 1 + denoted weak, incomplete staining in >10%; 2 + referred to moderate staining in >10% or strong staining in < 10%; and 3 + indicated strong, complete membranous staining in ≥ 10%. Tumors with a 2 + score underwent additional evaluation by fluorescence in situ hybridization (FISH) [13, 14].

The detailed inclusion and exclusion criteria for the Polish reimbursement access program are outlined in Supplementary Materials 1 [15]. Data collection encompassed initial patient weight, SG dosing, and (where applicable) reasons for initiating treatment with a reduced dose. Additional information included baseline demographic and clinical variables such as age, sex, performance status (PS), histological subtype, sites of metastases, *Breast Cancer* (*BRCA)* gene mutation status (if available), CPS (when assessed), and the number of prior systemic therapy lines. Treatment-related data covered the number of SG cycles administered, use of granulocyte colony-stimulating factor (G-CSF), any dose modifications or delays, and reasons for discontinuation. Adverse events were graded according to the National Cancer Institute Common Terminology Criteria for Adverse Events (NCI-CTCAE), version 5.0. Radiologic response was evaluated based on Response Evaluation Criteria in Solid Tumors (RECIST) version 1.1.

Data regarding real-world outcomes for SG safety and efficacy in Polish patients were reported in our previous publications [16–19].

## Study design

This ambispective, multicenter cohort study was conducted across four oncology centers in Poland and included both retrospective and prospective data from patients who had initiated the treatment with SG by 30th September 2024 outside the context of clinical trials. Eligibility required completion of at least one full treatment cycle (administration on days 1 and 8). Data collection was finalized as of 30 January 2025.

The study was conducted in accordance with the Good Reporting of Outcomes in Real-world Evidence Studies (GROW) guidelines, ensuring transparency and methodological rigor. Key aspects of real-world data methodology were addressed, including study design, patient selection criteria, data acquisition procedures, strategies for handling missing information, and systematic assessment of treatment safety [20].

## Ethical considerations

The study was approved by the ethics committees of the Maria Sklodowska-Curie National Research Institute of Oncology in Krakow (2/2023, 18 April 2023) and Warsaw (21/2024, 22 February 2024). All procedures followed institutional and Helsinki Declaration standards. Written informed consent was obtained before SG treatment; consent for retrospective data analysis was waived.

## Statistical considerations

Statistical analysis was performed using PS Imago Pro 9 software. Descriptive statistics were used to summarize baseline characteristics, treatment modalities, and the incidence of AEs. Continuous variables were reported as means with standard deviations (SD) or as medians with interquartile ranges (IQR), depending on data distribution. Categorical variables were presented as absolute numbers and percentages. Comparisons between groups for categorical variables were conducted using the chi-square test or Fisher’s exact test, as appropriate. PFS and OS were estimated using the Kaplan–Meier method, and survival curves were compared using log-rank tests based on selected clinical factors. Associations between clinical variables and survival outcomes were assessed using univariate Cox proportional hazards regression models. Variables with clinical relevance were further included in multivariate Cox models. A p-value < 0.05 was considered statistically significant.

## Results

### Patient demographics and clinical characteristics

As of 30 September 2024, SG therapy was initiated in 83 women with mTNBC across four oncology centers. The median age at treatment initiation was 55 years (interquartile range [IQR]: 46–65 years, range: 30–86 years). The mean Ki-67 proliferation index was 55.2% (standard deviation: 24.6; range: 5.0–100.0). A total of 25 patients (30.1%) had a prior diagnosis of hormone receptor-positive and/or HER2-positive breast cancer. At baseline, all patients had confirmed distant metastases. The median number of prior systemic therapy lines in the palliative setting was 2. Thirty-one patients had additionally received systemic treatment in the (neo)adjuvant setting. The median number of SG cycles completed was 4.

Further details on sociodemographic characteristics and prior therapies are presented in Table 1.

**Table 1:** Patient clinical characteristics

Parameter		Total (*N* = 83)
Stage at cancer diagnosis*	I-III	73 (88%)
	IV	10 (12%)
Comorbidities**	No	39 (47%)
	Yes	44 (53%)
Menopausal status	Premenopausal	24 (29%)
	Postmenopausal	59 (71%)
Site of metastatic disease at SG initiation	Lymph nodes	59 (71%)
	Lungs	41 (49%)
	Bones	35 (42%)
	Skin/subcutaneous tissue	32 (39%)
	Liver	26 (31%)
	Malignant effusion	10 (12%)
	Brain	7 (8%)
	Other	8 (10%)

*According to American Joint Committee on Cancer’s Staging System (8th edition)

**Comorbidities requiring active management and deemed clinically significant by the treating physician

Based on the dataset, the majority of patients (81%, 67/83) received cytotoxic chemotherapy as part of (neo)adjuvant systemic treatment. Hormonal therapy and anti-HER2 agents were administered in 18% (15/83) and 7% (6/83) of patients, respectively. Radical surgery had been performed in 81% (67/83) of the cohort, and 67% (56/83) had received radical radiotherapy.

Prior to SG treatment, patients were exposed to various palliative systemic therapies. Platinum-based regimens were the most common, administered to 42 patients (51%), followed by taxane-based regimens in 33 patients (40%) and anthracycline-based protocols in 20 patients (24%). Capecitabine was used in 25 patients (30%), gemcitabine in 23 (28%), vinorelbine in 13 (16%), hormonal agents in 10 (12%), pembrolizumab in 4 (5%), and PARP inhibitors in 4 (5%). Other systemic therapies were reported in 18 patients (22%).

Adverse events of grade ≥ 2 related to prior systemic treatments included neutropenia in 25 patients (30%), anemia in 24 (29%), elevated liver enzymes (ALT/AST) in 16 (19%), thrombocytopenia in 9 (11%), nausea and vomiting in 9 (11%) each, diarrhea in 6 (7%), and hypersensitivity reactions in 2 patients (2%). Febrile neutropenia and cardiac toxicity occurred in one patient each (1%).

## Sacituzumab Govitecan dosing and reasons for dose reduction

Initial dose reductions of SG ≥ 10% were observed in 16 patients (19.3%), including 9 patients (10.8%) who received a dose reduced by ≥ 20%. Administrative adjustments—defined as reductions > 10% to flat doses of 200, 400, 600, or 800 mg—accounted for 18.1% of the entire cohort. Other reported reasons for dose modification included anticipated or pre-existing toxicities, such as diarrhea or neutropenia.

Overall, dose reductions due to toxicity were required in 24.1% of patients after the treatment was initiated and treatment delays occurred in 68.7%.

## Treatment safety

The safety profile of SG was characterized primarily by hematologic toxicity, with most grade ≥ 3 AEs occurring within the first two to three cycles. The most common grade ≥ 3 AE was neutropenia, observed in 38 patients (45.8%), with a median onset in cycle 2. Other frequent grade ≥ 3 hematologic events included anemia in 7 patients (8.4%) and thrombocytopenia in 5 patients (6.0%), with median onset in cycles 2 and 1, respectively. Elevated liver enzymes of grade ≥ 3 were reported in 4 patients (4.8%), most commonly in cycle 3. Gastrointestinal toxicities of grade ≥ 3 were less common: diarrhea occurred in 3 patients (3.6%), nausea and hypersensitivity reactions each in 1 patient (1.2%), and no cases of grade ≥ 3 vomiting were reported. Hypersensitivity reactions and alopecia (any grade) were noted in 1.2% and 31.3% of patients, respectively.

The use of G-CSF was common: 85.5% of patients received some form of G-CSF support (including both: primary and secondary prophylaxis).

### The impact of dose reduction on treatment safety and efficacy

**Fig. 1:**
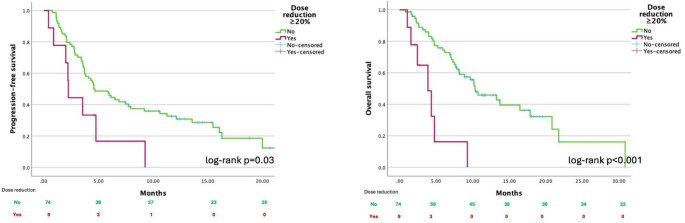
Progression-free survival and overall survival according to initial sacituzumab govitecan dose reduction ≥ 20% versus < 20%

In patients who received an initial SG dose reduction of ≥ 10% (*n* = 16), no statistically significant differences in PFS or OS were observed compared to those with < 10% dose reduction (log-rank *p* = 0.3 and *p* = 0.09, respectively), although a trend toward worse outcomes was noted (Supplementary materials, Fig. 1S). In contrast, patients who underwent an initial dose reduction ≥ 20% (*n* = 9) showed significantly shorter PFS (log-rank *p* = 0.03) and OS (log-rank *p* < 0.001), as demonstrated in Fig. 1. The median PFS in the < 20% dose-reduced group was 4.4 months (95% confidence interval [CI]: 2.6–6.5), compared to 2.2 months (95% CI: 2.1–2.3) in those with a ≥ 20% initial dose reduction. The corresponding OS medians were 10.3 months (95% CI: 6.8–13.8) and 3.9 months (95% CI: 1.7–6.0), respectively.

In univariate Cox regression analysis, patients with a ≥ 20% initial dose reduction had a twofold higher risk of disease progression (hazard ratio [HR]: 2.3; 95% CI: 1.1–4.9; *p* = 0.03) and a fivefold higher risk of death (HR: 4.8; 95% CI: 2.1–11.3; *p* < 0.001). After adjustment for clinically relevant factors (PS, age ≥ 65 years, comorbidities, line of SG treatment, and presence of brain metastases) in a multivariate Cox regression model, a ≥ 20% initial dose reduction remained an independent predictor of both shorter PFS (HR: 2.6; 95% CI: 1.1–6.6; *p* = 0.04) and OS (HR: 6; 95% CI: 2–17.5; *p* = 0.001) with detailed results in Table S1 in Supplementary Materials.

The initial dose reduction ≥ 20% had no statistically significant impact on the incidence of AEs ≥ grade 2 (*n* = 3, 66.7% vs. *n* = 63, 85.1%; *p* = 0.17), AEs ≥ grade 3 (*n* = 4, 44.4% vs. *n* = 43, 58.1%; *p* = 0.49), or on the frequency of G-CSF use (*n* = 8, 88.9% vs. *n* = 62, 83.8%; *p* = 1.00). Similarly, the rates of all-grade hematologic toxicities were not significantly different between these groups. Neutropenia occurred in 55.6% (*n* = 5) of patients in the ≥ 20% dose-reduction group vs. 64.9% (*n* = 48) in the < 20% group (*p* = 0.72), anemia in 33.3% (*n* = 3) vs. 36.5% (*n* = 27; *p* = 1.00), and thrombocytopenia in 11.1% (*n* = 1) vs. 12.2% (*n* = 9; *p* = 1.00), respectively.

## Discussion

In the phase 3 ASCENT trial, SG, administered at 10 mg/kg given on days 1 and 8 of a 21-day cycle, demonstrated a clear clinical benefit in patients with heavily pretreated metastatic TNBC, resulting in substantial improvements in both PFS and OS. The median PFS was 5.6 months and the median OS was 12.1 months, notably outperforming standard chemotherapy, which achieved median values of 1.7 months and 6.7 months, respectively [3].

This dosing regimen had been selected based on earlier clinical development, during which various SG dose levels were investigated [21]. The final results of the IMMU-132-01 trial—a phase 1/2, open-label, basket-design study involving patients with previously treated solid tumors—supported 10 mg/kg as a clinically feasible dose in the metastatic TNBC setting [22]. Although hematologic toxicity, particularly neutropenia, was more frequent at 10 mg/kg compared to 8 mg/kg, adverse events were generally manageable with supportive care and protocol-guided dose modifications. Moreover, exposure–response modeling in TNBC further demonstrated a clear association between higher SG exposure and improved clinical outcomes, including objective response rate and survival [23].

These findings provided the basis for the adoption of 10 mg/kg as the standard dosing strategy in clinical trials and regulatory approvals. However, despite this evidence-based standard, the extent to which it is applied in real-world clinical practice appears to vary considerably.

Real-world evidence demonstrates notable variability in the initial dosing of SG across clinical settings. A single-center German cohort reported that 8 of 43 patients (18.6%) began SG treatment at a reduced dose of 7.5 mg/kg, based solely on physician judgment. Although this approach likely reflects concerns about frailty or comorbidities, no comparative safety or efficacy outcomes were reported for this subgroup [24]. Similarly, in a multicenter study conducted across 16 tertiary cancer centers in the United Kingdom involving 132 patients with metastatic TNBC, 9% (12 patients) initiated treatment with SG at a reduced dose [25].

In contrast, real-world study conducted in the United States using ConcertAI Patient360™ database evaluated 230 patients with metastatic TNBC treated with SG in the second-line setting or later [26]. Among 170 patients with available dosing data, the median starting dose was 10.0 mg/kg (IQR: 9.8–10.1), indicating that nearly all patients initiated therapy at the full recommended dose, with few, if any, receiving clinically significant upfront dose reductions [26]. In another real-world study from the United States, approximately 10% of patients initiated SG at a reduced starting dose [27]. The median starting dose across the cohort was 10 mg/kg, with a range of 5–10 mg/kg. While no statistically significant association was observed between starting dose and objective response rate (ORR) or clinical benefit rate (CBR), a numerical trend suggested that patients with shorter duration of response tended to have received lower initial doses [27].

The issue of initiating SG at reduced doses remains controversial. In a recent Delphi consensus involving 51 Italian oncology experts, the panel broadly agreed that SG should be administered at the full recommended dose during the first cycle, with rare exceptions, as early dose reductions could compromise efficacy [8]. Upfront dose reduction was not deemed mandatory even for patients with the homozygous UGT1A1 *28/*28 genotype, and consensus was not reached on dose reduction in frail or elderly patients due to insufficient supporting evidence. Importantly, suggestions to adopt non–evidence-based upfront dose reductions—especially in populations not well represented in pivotal trials—failed to achieve consensus in two rounds of voting [8]. These results reflect a clear tendency toward starting SG at the standard dose, with dose modifications guided by real-time tolerability rather than precautionary assumptions.

Although expert consensus tends to support full-dose initiation, real-world evidence suggests that reduced starting doses may be associated with earlier treatment discontinuation and potentially diminished clinical benefit. In a cohort of over 400 patients treated with SG demonstrated that those who initiated therapy at 10 mg/kg had a significantly longer median duration on treatment compared to those who started at lower doses (136 vs. 77 days; *p* = 0.012) [28]. Interestingly, patients who underwent dose reductions during treatment had a longer median duration on therapy than those who did not (172 vs. 89 days; *p* < 0.001), suggesting that reactive dose modifications may support treatment continuity [28]. Although counterintuitive, this finding likely reflects that patients requiring dose reductions—due to manageable toxicity—can often continue therapy, whereas those without reductions may include individuals who discontinued early due to rapid disease progression. Collectively, these data support the strategy of initiating SG at the standard 10 mg/kg dose, with subsequent individualized dose adjustments based on patient tolerability, to maximize treatment exposure and clinical benefit.

In line with these findings, we observed that initiating treatment at a reduced dose was associated with shorter PFS and OS. It is also important to consider that shorter treatment duration may, at least in part, reflect unfavorable baseline characteristics such as reduced PS or the presence of brain metastases, that are themselves associated with poorer outcomes. Therefore, confirmation of these associations through multivariate analysis, as performed in this study, is essential to account for potential confounding variables.

Importantly, initial dose reductions are often based on physician discretion or empirical judgment in the absence of toxicity, reflecting a precautionary rather than evidence-based approach. In contrast, on-treatment dose reductions are typically driven by observed toxicities and are guided by clinical protocols and prescribing information. Thus, a reduction from 10 mg/kg to 8 mg/kg due to AEs during therapy is not equivalent (clinically or biologically) to initiating therapy at 8 mg/kg. The superior duration on treatment observed in patients managed through adaptive dose modifications rather than reduced starting doses suggests that real-time tailoring of treatment based on individual toxicity profiles is a more effective strategy for maintaining patients on SG and optimizing clinical benefit.

This distinction is further supported by trial data. Detailed safety analyses from the ASCENT trial showed that dose reductions of SG, implemented reactively in 22% of patients in response to toxicity, did not compromise treatment efficacy [29]. Despite these modifications, SG maintained its clinical benefit, with ORR, clinical benefit rate (CBR), and PFS remaining superior to treatment of physician’s choice across subgroups, as reflected by median PFS values of 8.3 vs. 2.9 months in patients with dose reductions, and 4.6 vs. 1.5 months in those without [29].

Nevertheless, the decision to initiate SG at a reduced starting dose is influenced by a complex interplay of regulatory guidance, reimbursement policies, and clinical judgment. SG received FDA approval on April 7, 2021, for patients with unresectable locally advanced or metastatic TNBC after two or more prior systemic therapies, including at least one in the metastatic setting [30]. On February 3, 2023, it was also approved for HR-positive, HER2-negative metastatic BC after progression on endocrine therapy and at least two additional lines of treatment [31]. SG previously held accelerated approval for metastatic urothelial cancer following prior treatment with a platinum-containing chemotherapy and a programmed death receptor-1 (PD-1) or PD-L1 inhibitor, granted in April 2021. However, this indication was withdrawn by the FDA on November 22, 2024, following negative confirmatory data [32].

Despite its evolving regulatory history, the Summary of Product Characteristics for SG does not include any information regarding the possibility of initiating treatment at a reduced dose [12]. Although the product label provides clear recommendations for dose modifications in response to treatment-emergent toxicities, it does not endorse routine upfront dose reductions based solely on physician judgment or perceived patient frailty. This disconnect reveals a potential misalignment between current clinical practice and the approved dosing guidance issued by the European Medicines Agency, raising important questions about how best to reconcile individualized clinical decision-making with standardized regulatory frameworks.

At the same time, national reimbursement policies may have a significant impact on how high-cost anticancer drugs are initially dosed, especially when dosing is based on body weight or surface area. Such dosing requires individualized preparation from fixed vial sizes, often resulting in leftover drug that must be discarded due to short post-reconstitution stability. The financial impact of this wastage is substantial. A U.S. analysis of the top 20 cancer drugs packaged in single-use vials estimated that $1.8 billion—approximately 10% of $18 billion in projected 2016 revenue—was attributable to discarded drug alone, depending on available vial sizes and market practices [33]. In Poland, SG is reimbursed through a national drug program that covers only the amount of drug actually administered; any unused portion of a vial is not reimbursed. This creates a financial constraint to administering full doses when doing so would require opening an additional vial for a marginal excess beyond the available vial size (e.g., >200 mg). There is an urgent need for reimbursement models that reconcile economic efficiency with evidence-based dosing to ensure optimal patient care.

One proposed solution to mitigate drug waste while preserving therapeutic integrity is rational dose optimization using pharmacokinetic modeling. A recent modeling study developed a weight-band–based dosing strategy for SG, aiming to minimize drug waste and financial burden while preserving systemic exposure equivalent to the standard 10 mg/kg dose [34]. Using the license holder’s published pharmacokinetic model, the authors demonstrated that alternative dosing could lower drug use by approximately 20%, with geometric mean ratios for area under the concentration-time curve, trough concentration and maximum concentration falling within the predefined equivalence boundaries (0.90–1.11). Such approach may offer a systematic framework for reducing drug waste.

Nonetheless other factors, such as physician concerns regarding patient frailty, prior toxicities, or entrenched institutional habits remain underexplored. A key gap in current evidence is the lack of qualitative data exploring the clinical rationale behind initial dose reductions of SG in routine practice. Understanding these contextual factors represents an important avenue for future research and may help inform more nuanced, evidence-based dosing guidelines [8, 24, 28].

Moreover, the relationship between initial dose reduction of SG and treatment tolerability remains unclear [28], and is often insufficiently explored. Several real-world studies did not systematically assess whether lower starting doses were associated with reduced toxicity [24, 25]. While some clinicians adopt a precautionary approach based on perceived risk, available data suggest that this strategy may not consistently improve safety outcomes. Further studies are warranted to differentiate the clinical impact of proactive (preemptive) versus reactive (toxicity-driven) dose reductions, particularly in terms of their influence on treatment efficacy, tolerability, and overall outcomes.

This study has several limitations inherent to its ambispective, observational design. First, the sample size was relatively small, particularly within the subgroup of patients receiving substantial initial dose reductions (≥ 20%), which may limit the generalizability and statistical power of subgroup analyses. As a result, we did not perform a direct comparison of demographic or baseline clinical characteristics between patients who received an initial dose reduction ≥ 20% and those who did not. Instead, multivariate Cox regression models were used to adjust for clinically relevant factors and account for potential confounding. While this analytical approach strengthens the validity of the observed associations, the small number of patients in the high-reduction group (*n* = 9) limits the precision of effect estimates and precludes more granular subgroup comparisons. The effect of primary vs. secondary G-CSF implementation was not evaluated for this study and only overall use was recorded. We acknowledge this as a limitation of our findings and plan to address it through an expanded prospective cohort currently in development.

Despite adjustment for known covariates, the non-randomized nature of the study means that residual confounding cannot be entirely excluded—particularly as the decision to initiate SG at a reduced dose was made at the discretion of treating physicians and may have been influenced by undocumented clinical, logistical, or institutional factors. The study did not include formal patient-reported outcomes or health-related quality-of-life assessments, which may be important for fully understanding the implications of dose modifications from the patient perspective. Finally, treatment delays and adverse event reporting were based on routine clinical documentation and were not prospectively adjudicated, which may have introduced reporting bias. Despite these limitations, the multicenter design, real-world setting, and adherence to Good Reporting of Outcomes in Real-world Evidence Studies methodology enhance the external validity, transparency, and clinical applicability of the study findings.

## Conclusions

Initiating sacituzumab govitecan at a substantially reduced starting dose (≥ 20%) may compromise clinical efficacy without offering a meaningful safety advantage. In this multicenter real-world cohort of patients with metastatic triple-negative breast cancer, such dose attenuation was associated with significantly shorter progression-free and overall survival, while rates of adverse events and supportive treatment needs remained comparable. In contrast, dose reductions due to toxicity did not seem to compromise treatment duration or outcomes. This underscores the value of a patient-tailored, adaptive dosing approach. The findings support initiating SG at the full recommended dose of 10 mg/kg, with subsequent modifications guided by individual tolerability. Further prospective research is needed to validate these results and to develop dosing strategies that balance efficacy, safety, and economic feasibility in routine clinical practice.

## Supplementary Information

Below is the link to the electronic supplementary material.

Supplementary Material 1

## Data Availability

Data generated and analyzed in this study are available from the corresponding author upon reasonable request.
