# Supplementary material for: Sacituzumab Govitecan initial dose reduction in polish patients with metastatic triple-negative breast cancer: impact on efficacy and safety
Source: Cancer Chemother Pharmacol. 2025 Oct 4;95(1):97. doi: 10.1007/s00280-025-04823-3 (PMC12496269; doi:10.1007/s00280-025-04823-3)
Supplement: Supplementary file 1 — Supplementary Material 1 [file 280_2025_4823_MOESM1_ESM.docx]

**Table S1.** Multivariate Cox regression model for progression-free survival and overall survival with clinically relevant factors identified as potential confounders.

|  | Hazard ratio | 95% CI | p-value |
| --- | --- | --- | --- |
| Progression-free survival, p=0.3 | | | |
| SG dose reduction ≥20% | 2.6 | 1.1-6.6 | 0.03* |
| Age≥65 years | 0.6 | 0.3-1.1 | 0.1 |
| ECOG performance status | 1.1 | 0.7-2 | 0.6 |
| Line of SG treatment | 1.1 | 0.8-1.3 | 0.6 |
| Presence of brain metastases | 0.9 | 0.4-2.3 | 0.9 |
| Comorbidities** | 1 | 0.5-1.7 | 0.9 |
| Overall survival, p=0.02* | | | |
| SG dose reduction ≥20% | 6 | 2-17.5 | 0.001* |
| Age≥65 years | 1.1 | 0.6- 2.1 | 0.7 |
| ECOG performance status | 1.7 | 0.8- 3.5 | 0.1 |
| Line of SG treatment | 1 | 0.8- 1.4 | 0.8 |
| Presence of brain metastases | 0.8 | 0.2-2.4 | 0.7 |
| Comorbidities** | 0.6 | 0.3-1.3 | 0.2 |

Abbreviations: CI- confidence interval, ECOG- Eastern Cooperative Oncology Group, SG- sacitizumab govitecan.

*- values statistically significant
**- comorbidities requiring active management and deemed clinically significant by the treating physician.


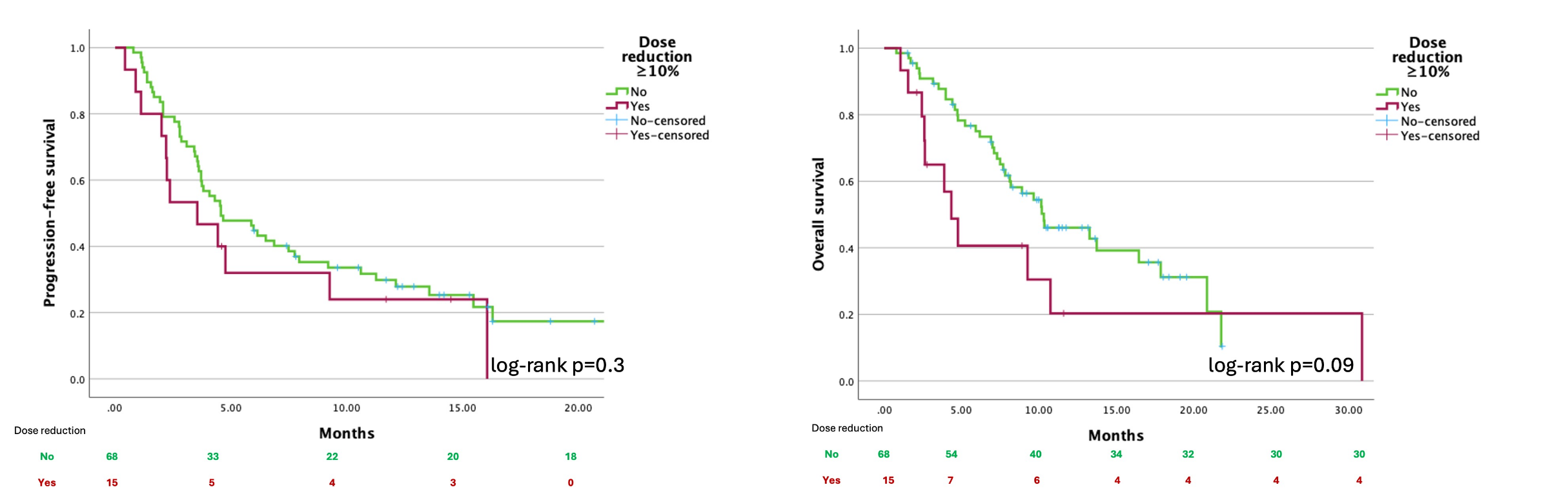


**Figure S1.** Progression-free survival and overall survival in patients with initial sacituzumab govitecan dose reduction ≥10% versus <10%.
